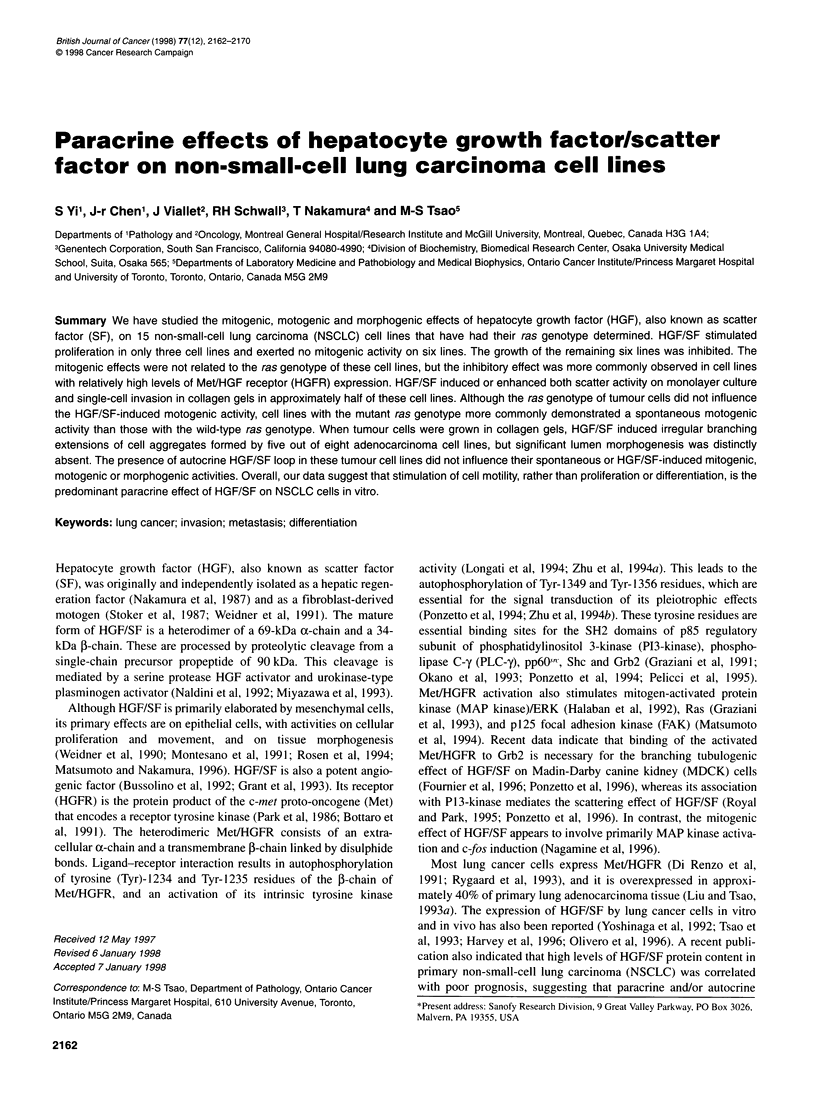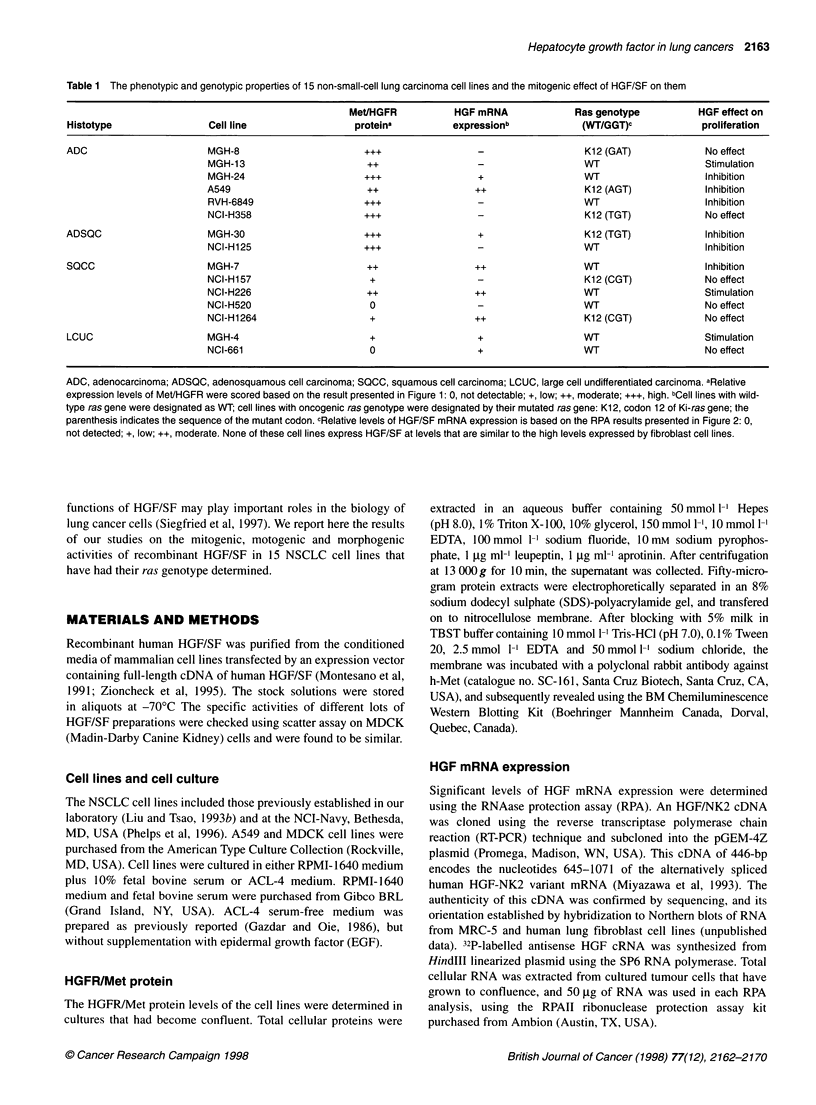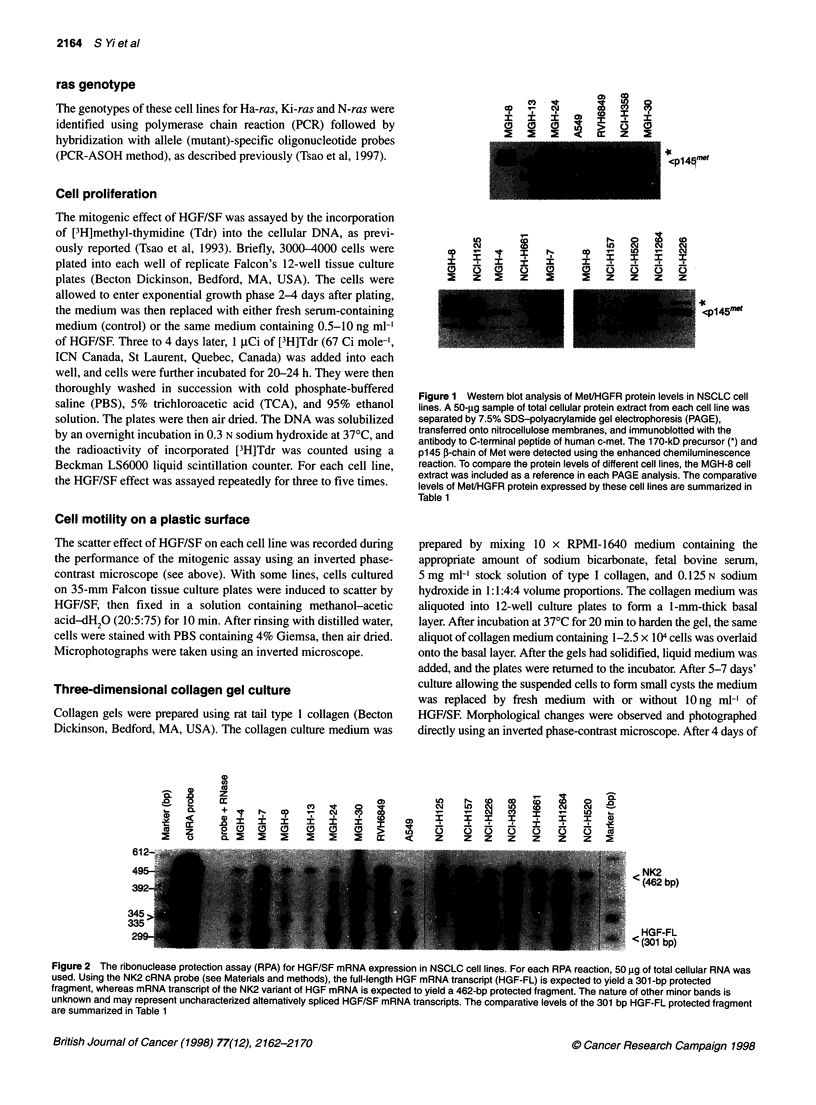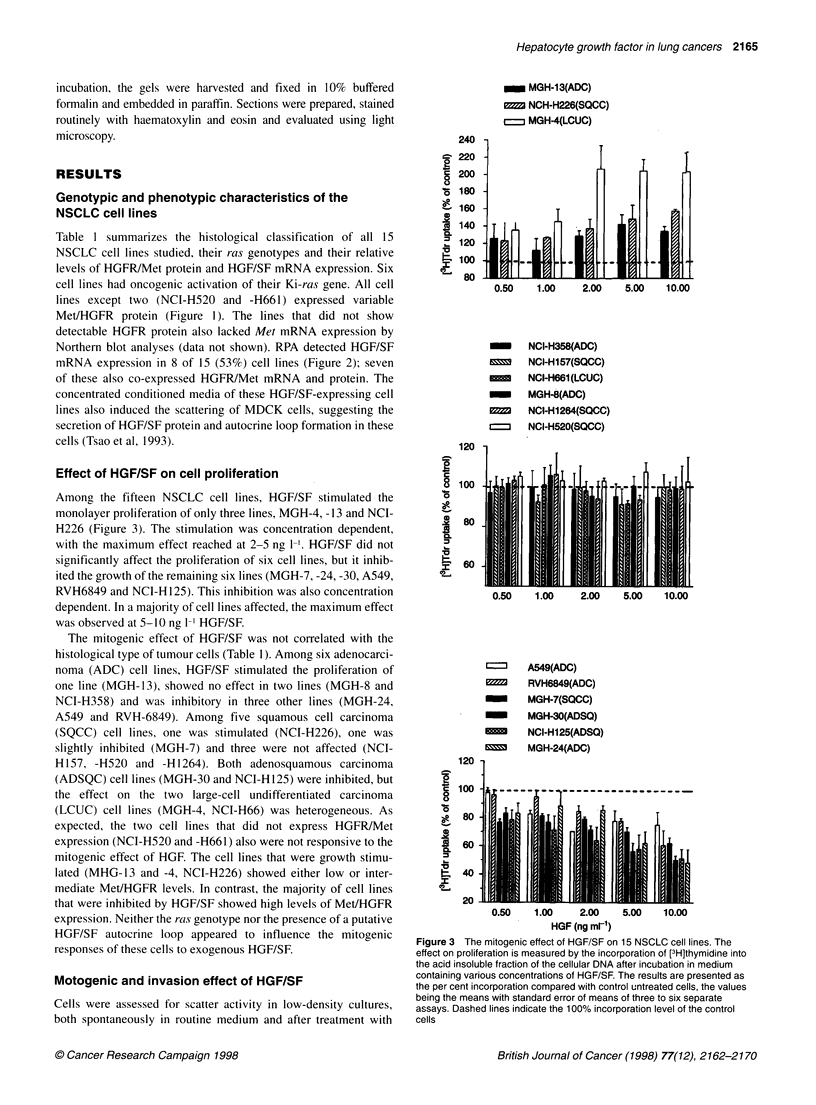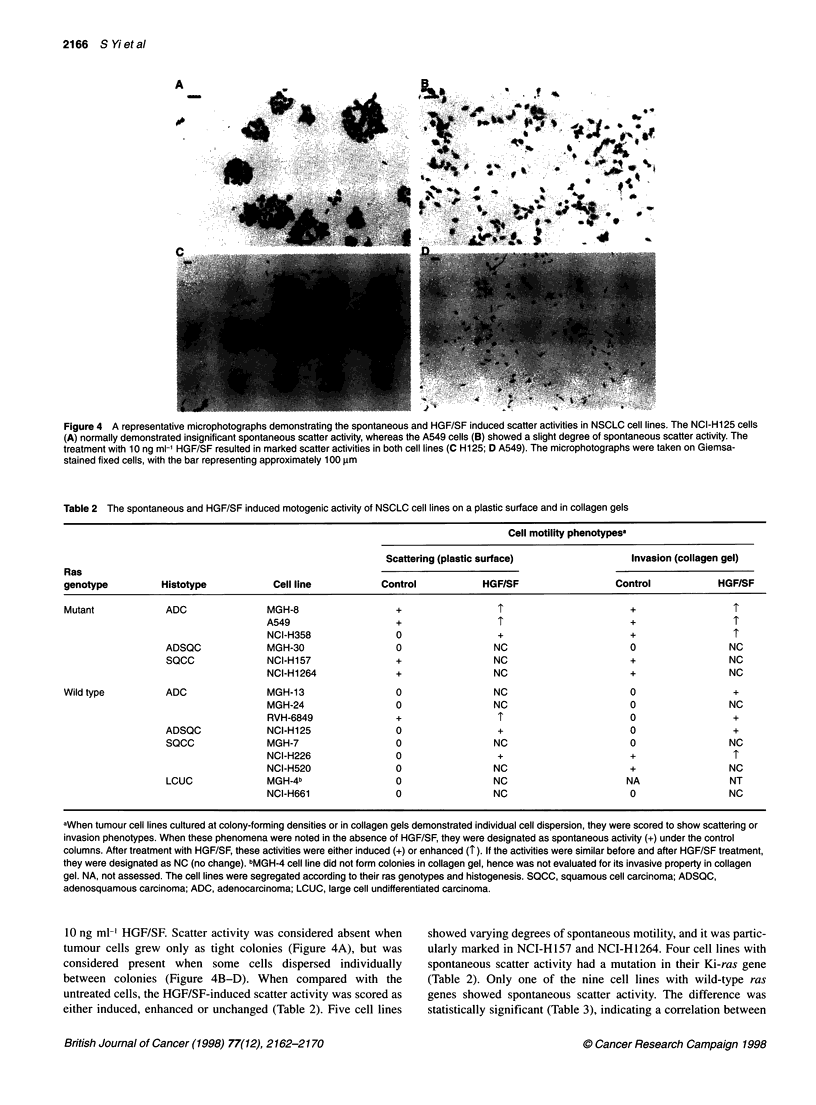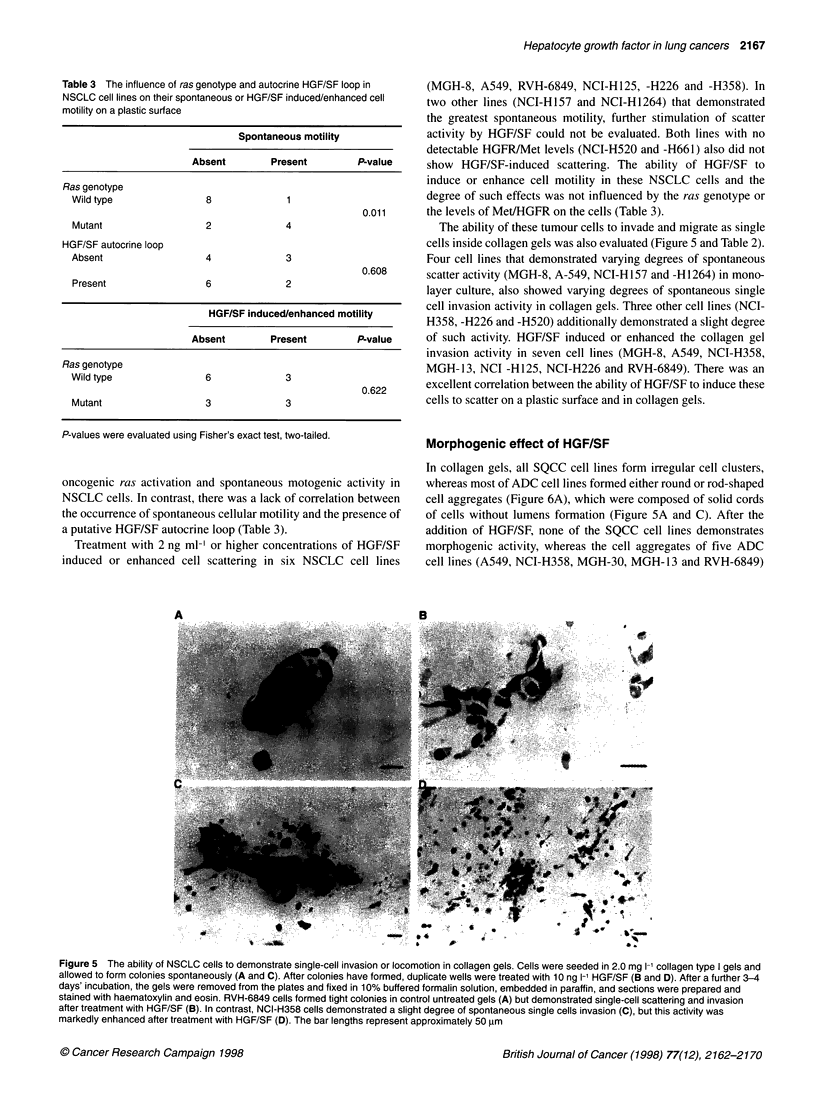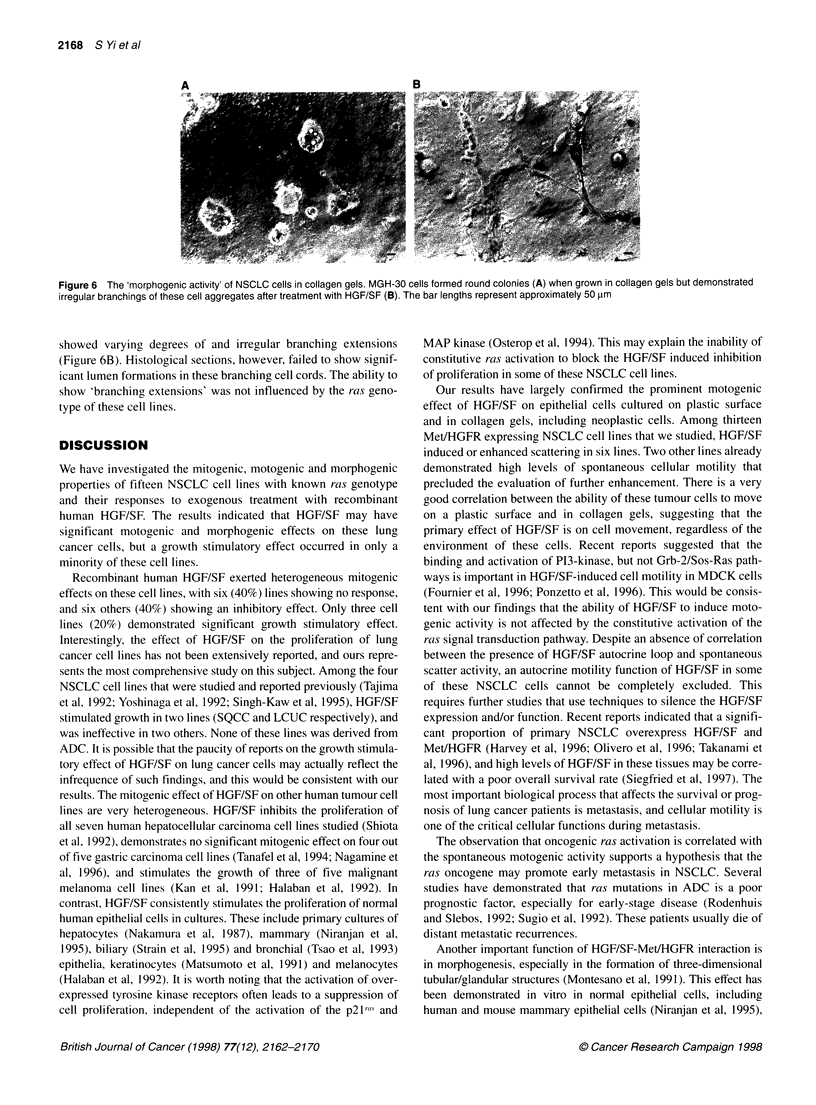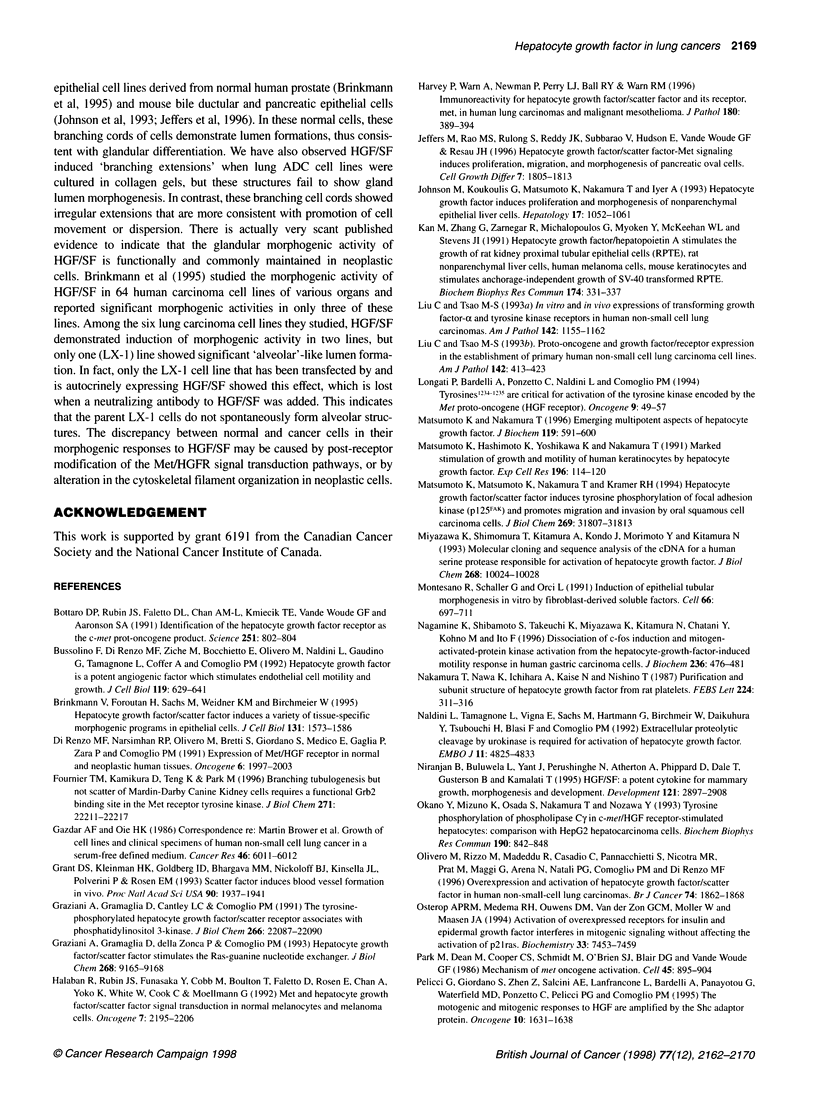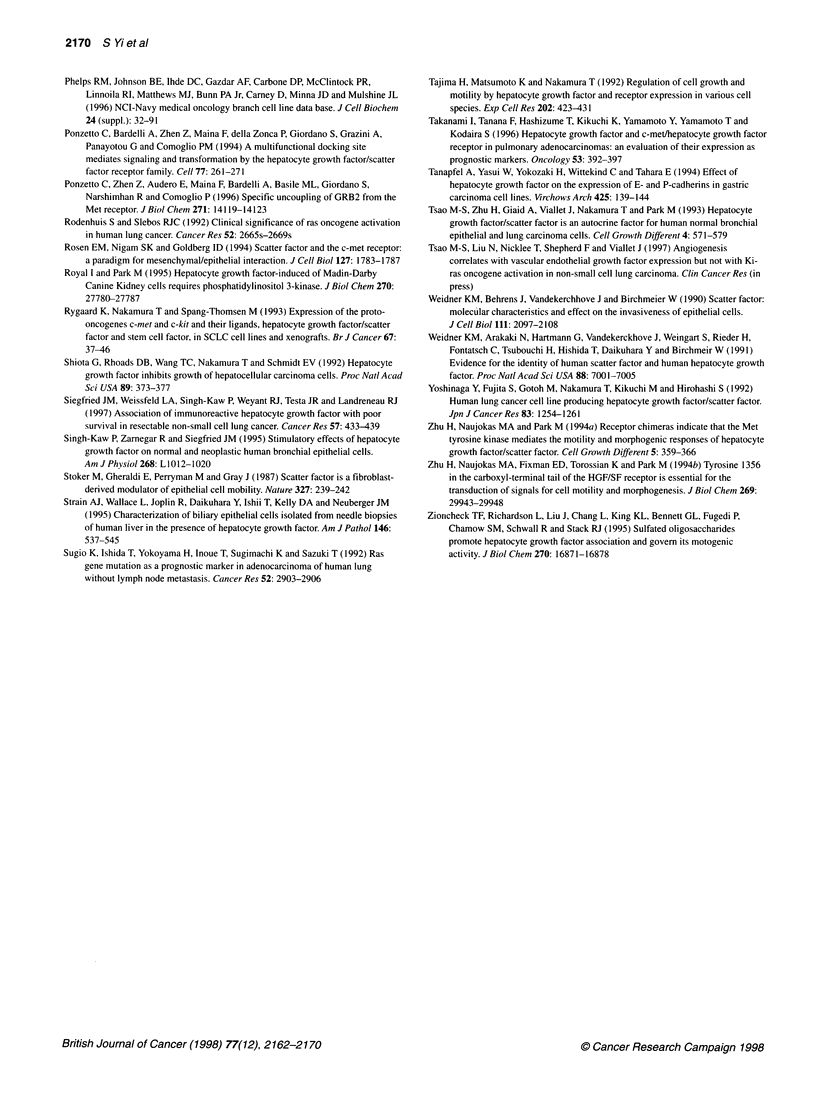# Paracrine effects of hepatocyte growth factor/scatter factor on non-small-cell lung carcinoma cell lines.

**DOI:** 10.1038/bjc.1998.361

**Published:** 1998-06

**Authors:** S. Yi, J. R. Chen, J. Viallet, R. H. Schwall, T. Nakamura, M. S. Tsao

**Affiliations:** Department of Pathology, Montreal General Hospital/Research Institute and McGill University, Montreal, Quebec, Canada.

## Abstract

**Images:**


British Journal of Cancer (1998) 77(12), 2162–2170
© 1998 Cancer Research Campaign

### Paracrine effects of hepatocyte growth factor/scatter factor on non-small-cell lung carcinoma cell lines

**S Yi[1], J-r Chen[1], J Viallet[2], RH Schwall[3], T Nakamura[4] and M-S Tsao[5]**

Departments of [1]Pathology and [2]Oncology, Montreal General Hospital/Research Institute and McGill University, Montreal, Quebec, Canada H3G 1A4; [3]Genentech Corporation, South San Francisco, California 94080-4990; [4]Division of Biochemistry, Biomedical Research Center, Osaka University Medical School, Suita, Osaka 565; [5]Departments of Laboratory Medicine and Pathobiology and Medical Biophysics, Ontario Cancer Institute/Princess Margaret Hospital and University of Toronto, Toronto, Ontario, Canada M5G 2M9

**Summary** We have studied the mitogenic, motogenic and morphogenic effects of hepatocyte growth factor (HGF), also known as scatter factor (SF), on 15 non-small-cell lung carcinoma (NSCLC) cell lines that have had their *ras* genotype determined. HGF/SF stimulated proliferation in only three cell lines and exerted no mitogenic activity on six lines. The growth of the remaining six lines was inhibited. The mitogenic effects were not related to the *ras* genotype of these cell lines, but the inhibitory effect was more commonly observed in cell lines with relatively high levels of Met/HGF receptor (HGFR) expression. HGF/SF induced or enhanced both scatter activity on monolayer culture and single-cell invasion in collagen gels in approximately half of these cell lines. Although the *ras* genotype of tumour cells did not influence the HGF/SF-induced motogenic activity, cell lines with the mutant *ras* genotype more commonly demonstrated a spontaneous motogenic activity than those with the wild-type *ras* genotype. When tumour cells were grown in collagen gels, HGF/SF induced irregular branching extensions of cell aggregates formed by five out of eight adenocarcinoma cell lines, but significant lumen morphogenesis was distinctly absent. The presence of autocrine HGF/SF loop in these tumour cell lines did not influence their spontaneous or HGF/SF-induced mitogenic, motogenic or morphogenic activities. Overall, our data suggest that stimulation of cell motility, rather than proliferation or differentiation, is the predominant paracrine effect of HGF/SF on NSCLC cells in vitro.

**Keywords:** lung cancer; invasion; metastasis; differentiation

Hepatocyte growth factor (HGF), also known as scatter factor (SF), was originally and independently isolated as a hepatic regeneration factor (Nakamura et al, 1987) and as a fibroblast-derived motogen (Stoker et al, 1987; Weidner et al, 1991). The mature form of HGF/SF is a heterodimer of a 69-kDa α-chain and a 34-kDa β-chain. These are processed by proteolytic cleavage from a single-chain precursor propeptide of 90 kDa. This cleavage is mediated by a serine protease HGF activator and urokinase-type plasminogen activator (Naldini et al, 1992; Miyazawa et al, 1993).

Although HGF/SF is primarily elaborated by mesenchymal cells, its primary effects are on epithelial cells, with activities on cellular proliferation and movement, and on tissue morphogenesis (Weidner et al, 1990; Montesano et al, 1991; Rosen et al, 1994; Matsumoto and Nakamura, 1996). HGF/SF is also a potent angiogenic factor (Bussolino et al, 1992; Grant et al, 1993). Its receptor (HGFR) is the protein product of the c-*met* proto-oncogene (Met) that encodes a receptor tyrosine kinase (Park et al, 1986; Bottaro et al, 1991). The heterodimeric Met/HGFR consists of an extracellular α-chain and a transmembrane β-chain linked by disulphide bonds. Ligand–receptor interaction results in autophosphorylation of tyrosine (Tyr)-1234 and Tyr-1235 residues of the β-chain of Met/HGFR, and an activation of its intrinsic tyrosine kinase activity (Longati et al, 1994; Zhu et al, 1994*a*). This leads to the autophosphorylation of Tyr-1349 and Tyr-1356 residues, which are essential for the signal transduction of its pleiotrophic effects (Ponzetto et al, 1994; Zhu et al, 1994*b*). These tyrosine residues are essential binding sites for the SH2 domains of p85 regulatory subunit of phosphatidylinositol 3-kinase (PI3-kinase), phospholipase C-γ (PLC-γ), pp60$^{src}$, Shc and Grb2 (Graziani et al, 1991; Okano et al, 1993; Ponzetto et al, 1994; Pelicci et al, 1995). Met/HGFR activation also stimulates mitogen-activated protein kinase (MAP kinase)/ERK (Halaban et al, 1992), Ras (Graziani et al, 1993), and p125 focal adhesion kinase (FAK) (Matsumoto et al, 1994). Recent data indicate that binding of the activated Met/HGFR to Grb2 is necessary for the branching tubulogenic effect of HGF/SF on Madin-Darby canine kidney (MDCK) cells (Fournier et al, 1996; Ponzetto et al, 1996), whereas its association with P13-kinase mediates the scattering effect of HGF/SF (Royal and Park, 1995; Ponzetto et al, 1996). In contrast, the mitogenic effect of HGF/SF appears to involve primarily MAP kinase activation and c-*fos* induction (Nagamine et al, 1996).

Most lung cancer cells express Met/HGFR (Di Renzo et al, 1991; Rygaard et al, 1993), and it is overexpressed in approximately 40% of primary lung adenocarcinoma tissue (Liu and Tsao, 1993*a*). The expression of HGF/SF by lung cancer cells in vitro and in vivo has also been reported (Yoshinaga et al, 1992; Tsao et al, 1993; Harvey et al, 1996; Olivero et al, 1996). A recent publication also indicated that high levels of HGF/SF protein content in primary non-small-cell lung carcinoma (NSCLC) was correlated with poor prognosis, suggesting that paracrine and/or autocrine

*Received 12 May 1997*
*Revised 6 January 1998*
*Accepted 7 January 1998*

*Correspondence to:* M-S Tsao, Department of Pathology, Ontario Cancer Institute/Princess Margaret Hospital, 610 University Avenue, Toronto, Ontario M5G 2M9, Canada

*Present address: Sanofy Research Division, 9 Great Valley Parkway, PO Box 3026, Malvern, PA 19355, USA

**Table 1** The phenotypic and genotypic properties of 15 non-small-cell lung carcinoma cell lines and the mitogenic effect of HGF/SF on them

| Histotype | Cell line | Met/HGFR protein[a] | HGF mRNA expression[b] | Ras genotype (WT/GGT)[c] | HGF effect on proliferation |
|---|---|---|---|---|---|
| ADC | MGH-8 | +++ | – | K12 (GAT) | No effect |
| | MGH-13 | ++ | – | WT | Stimulation |
| | MGH-24 | +++ | + | WT | Inhibition |
| | A549 | ++ | ++ | K12 (AGT) | Inhibition |
| | RVH-6849 | +++ | – | WT | Inhibition |
| | NCI-H358 | +++ | – | K12 (TGT) | No effect |
| ADSQC | MGH-30 | +++ | + | K12 (TGT) | Inhibition |
| | NCI-H125 | +++ | – | WT | Inhibition |
| SQCC | MGH-7 | ++ | ++ | WT | Inhibition |
| | NCI-H157 | + | – | K12 (CGT) | No effect |
| | NCI-H226 | ++ | ++ | WT | Stimulation |
| | NCI-H520 | 0 | – | WT | No effect |
| | NCI-H1264 | + | ++ | K12 (CGT) | No effect |
| LCUC | MGH-4 | + | + | WT | Stimulation |
| | NCI-661 | 0 | + | WT | No effect |

ADC, adenocarcinoma; ADSQC, adenosquamous cell carcinoma; SQCC, squamous cell carcinoma; LCUC, large cell undifferentiated carcinoma. [a]Relative expression levels of Met/HGFR were scored based on the result presented in Figure 1: 0, not detectable; +, low; ++, moderate; +++, high. [b]Cell lines with wild-type *ras* gene were designated as WT; cell lines with oncogenic *ras* genotype were designated by their mutated *ras* gene: K12, codon 12 of Ki-*ras* gene; the parenthesis indicates the sequence of the mutant codon. [c]Relative levels of HGF/SF mRNA expression is based on the RPA results presented in Figure 2: 0, not detected; +, low; ++, moderate. None of these cell lines express HGF/SF at levels that are similar to the high levels expressed by fibroblast cell lines.

functions of HGF/SF may play important roles in the biology of lung cancer cells (Siegfried et al, 1997). We report here the results of our studies on the mitogenic, motogenic and morphogenic activities of recombinant HGF/SF in 15 NSCLC cell lines that have had their *ras* genotype determined.

### MATERIALS AND METHODS

Recombinant human HGF/SF was purified from the conditioned media of mammalian cell lines transfected by an expression vector containing full-length cDNA of human HGF/SF (Montesano et al, 1991; Zioncheck et al, 1995). The stock solutions were stored in aliquots at –70°C The specific activities of different lots of HGF/SF preparations were checked using scatter assay on MDCK (Madin-Darby Canine Kidney) cells and were found to be similar.

### Cell lines and cell culture

The NSCLC cell lines included those previously established in our laboratory (Liu and Tsao, 1993*b*) and at the NCI-Navy, Bethesda, MD, USA (Phelps et al, 1996). A549 and MDCK cell lines were purchased from the American Type Culture Collection (Rockville, MD, USA). Cell lines were cultured in either RPMI-1640 medium plus 10% fetal bovine serum or ACL-4 medium. RPMI-1640 medium and fetal bovine serum were purchased from Gibco BRL (Grand Island, NY, USA). ACL-4 serum-free medium was prepared as previously reported (Gazdar and Oie, 1986), but without supplementation with epidermal growth factor (EGF).

### HGFR/Met protein

The HGFR/Met protein levels of the cell lines were determined in cultures that had become confluent. Total cellular proteins were extracted in an aqueous buffer containing 50 mmol $l^{-1}$ Hepes (pH 8.0), 1% Triton X-100, 10% glycerol, 150 mmol $l^{-1}$, 10 mmol $l^{-1}$ EDTA, 100 mmol $l^{-1}$ sodium fluoride, 10 mM sodium pyrophosphate, 1 μg $ml^{-1}$ leupeptin, 1 μg $ml^{-1}$ aprotinin. After centrifugation at 13 000 ***g*** for 10 min, the supernatant was collected. Fifty-microgram protein extracts were electrophoretically separated in an 8% sodium dodecyl sulphate (SDS)-polyacrylamide gel, and transfered on to nitrocellulose membrane. After blocking with 5% milk in TBST buffer containing 10 mmol $l^{-1}$ Tris-HCl (pH 7.0), 0.1% Tween 20, 2.5 mmol $l^{-1}$ EDTA and 50 mmol $l^{-1}$ sodium chloride, the membrane was incubated with a polyclonal rabbit antibody against h-Met (catalogue no. SC-161, Santa Cruz Biotech, Santa Cruz, CA, USA), and subsequently revealed using the BM Chemiluminescence Western Blotting Kit (Boehringer Mannheim Canada, Dorval, Quebec, Canada).

### HGF mRNA expression

Significant levels of HGF mRNA expression were determined using the RNAase protection assay (RPA). An HGF/NK2 cDNA was cloned using the reverse transcriptase polymerase chain reaction (RT-PCR) technique and subcloned into the pGEM-4Z plasmid (Promega, Madison, WN, USA). This cDNA of 446-bp encodes the nucleotides 645–1071 of the alternatively spliced human HGF-NK2 variant mRNA (Miyazawa et al, 1993). The authenticity of this cDNA was confirmed by sequencing, and its orientation established by hybridization to Northern blots of RNA from MRC-5 and human lung fibroblast cell lines (unpublished data). $^{32}$P-labelled antisense HGF cRNA was synthesized from *Hin*dIII linearized plasmid using the SP6 RNA polymerase. Total cellular RNA was extracted from cultured tumour cells that have grown to confluence, and 50 μg of RNA was used in each RPA analysis, using the RPAII ribonuclease protection assay kit purchased from Ambion (Austin, TX, USA).

© Cancer Research Campaign 1998

### ras genotype

The genotypes of these cell lines for Ha-*ras*, Ki-*ras* and N-*ras* were identified using polymerase chain reaction (PCR) followed by hybridization with allele (mutant)-specific oligonucleotide probes (PCR-ASOH method), as described previously (Tsao et al, 1997).

### Cell proliferation

The mitogenic effect of HGF/SF was assayed by the incorporation of [$^3$H]methyl-thymidine (Tdr) into the cellular DNA, as previously reported (Tsao et al, 1993). Briefly, 3000–4000 cells were plated into each well of replicate Falcon's 12-well tissue culture plates (Becton Dickinson, Bedford, MA, USA). The cells were allowed to enter exponential growth phase 2–4 days after plating, the medium was then replaced with either fresh serum-containing medium (control) or the same medium containing 0.5–10 ng ml$^{-1}$ of HGF/SF. Three to 4 days later, 1 μCi of [$^3$H]Tdr (67 Ci mole$^{-1}$, ICN Canada, St Laurent, Quebec, Canada) was added into each well, and cells were further incubated for 20–24 h. They were then thoroughly washed in succession with cold phosphate-buffered saline (PBS), 5% trichloroacetic acid (TCA), and 95% ethanol solution. The plates were then air dried. The DNA was solubilized by an overnight incubation in 0.3 N sodium hydroxide at 37°C, and the radioactivity of incorporated [$^3$H]Tdr was counted using a Beckman LS6000 liquid scintillation counter. For each cell line, the HGF/SF effect was assayed repeatedly for three to five times.

**Figure 1** Western blot analysis of Met/HGFR protein levels in NSCLC cell lines. A 50-μg sample of total cellular protein extract from each cell line was separated by 7.5% SDS–polyacrylamide gel electrophoresis (PAGE), transferred onto nitrocellulose membranes, and immunoblotted with the antibody to C-terminal peptide of human c-met. The 170-kD precursor (*) and p145 β-chain of Met were detected using the enhanced chemiluminescence reaction. To compare the protein levels of different cell lines, the MGH-8 cell extract was included as a reference in each PAGE analysis. The comparative levels of Met/HGFR protein expressed by these cell lines are summarized in Table 1

### Cell motility on a plastic surface

The scatter effect of HGF/SF on each cell line was recorded during the performance of the mitogenic assay using an inverted phase-contrast microscope (see above). With some lines, cells cultured on 35-mm Falcon tissue culture plates were induced to scatter by HGF/SF, then fixed in a solution containing methanol–acetic acid–d$H_2O$ (20:5:75) for 10 min. After rinsing with distilled water, cells were stained with PBS containing 4% Giemsa, then air dried. Microphotographs were taken using an inverted microscope.

### Three-dimensional collagen gel culture

Collagen gels were prepared using rat tail type 1 collagen (Becton Dickinson, Bedford, MA, USA). The collagen culture medium was prepared by mixing 10 × RPMI-1640 medium containing the appropriate amount of sodium bicarbonate, fetal bovine serum, 5 mg ml$^{-1}$ stock solution of type I collagen, and 0.125 N sodium hydroxide in 1:1:4:4 volume proportions. The collagen medium was aliquoted into 12-well culture plates to form a 1-mm-thick basal layer. After incubation at 37°C for 20 min to harden the gel, the same aliquot of collagen medium containing 1–2.5 × 10$^4$ cells was overlaid onto the basal layer. After the gels had solidified, liquid medium was added, and the plates were returned to the incubator. After 5–7 days' culture allowing the suspended cells to form small cysts the medium was replaced by fresh medium with or without 10 ng ml$^{-1}$ of HGF/SF. Morphological changes were observed and photographed directly using an inverted phase-contrast microscope. After 4 days of

**Figure 2** The ribonuclease protection assay (RPA) for HGF/SF mRNA expression in NSCLC cell lines. For each RPA reaction, 50 μg of total cellular RNA was used. Using the NK2 cRNA probe (see Materials and methods), the full-length HGF mRNA transcript (HGF-FL) is expected to yield a 301-bp protected fragment, whereas mRNA transcript of the NK2 variant of HGF mRNA is expected to yield a 462-bp protected fragment. The nature of other minor bands is unknown and may represent uncharacterized alternatively spliced HGF/SF mRNA transcripts. The comparative levels of the 301 bp HGF-FL protected fragment are summarized in Table 1

 © Cancer Research Campaign 1998

incubation, the gels were harvested and fixed in 10% buffered formalin and embedded in paraffin. Sections were prepared, stained routinely with haematoxylin and eosin and evaluated using light microscopy.

### RESULTS

### Genotypic and phenotypic characteristics of the NSCLC cell lines

Table 1 summarizes the histological classification of all 15 NSCLC cell lines studied, their *ras* genotypes and their relative levels of HGFR/Met protein and HGF/SF mRNA expression. Six cell lines had oncogenic activation of their Ki-*ras* gene. All cell lines except two (NCI-H520 and -H661) expressed variable Met/HGFR protein (Figure 1). The lines that did not show detectable HGFR protein also lacked *Met* mRNA expression by Northern blot analyses (data not shown). RPA detected HGF/SF mRNA expression in 8 of 15 (53%) cell lines (Figure 2); seven of these also co-expressed HGFR/Met mRNA and protein. The concentrated conditioned media of these HGF/SF-expressing cell lines also induced the scattering of MDCK cells, suggesting the secretion of HGF/SF protein and autocrine loop formation in these cells (Tsao et al, 1993).

### Effect of HGF/SF on cell proliferation

Among the fifteen NSCLC cell lines, HGF/SF stimulated the monolayer proliferation of only three lines, MGH-4, -13 and NCI-H226 (Figure 3). The stimulation was concentration dependent, with the maximum effect reached at 2–5 ng $l^{-1}$. HGF/SF did not significantly affect the proliferation of six cell lines, but it inhibited the growth of the remaining six lines (MGH-7, -24, -30, A549, RVH6849 and NCI-H125). This inhibition was also concentration dependent. In a majority of cell lines affected, the maximum effect was observed at 5–10 ng $l^{-1}$ HGF/SF.

The mitogenic effect of HGF/SF was not correlated with the histological type of tumour cells (Table 1). Among six adenocarcinoma (ADC) cell lines, HGF/SF stimulated the proliferation of one line (MGH-13), showed no effect in two lines (MGH-8 and NCI-H358) and was inhibitory in three other lines (MGH-24, A549 and RVH-6849). Among five squamous cell carcinoma (SQCC) cell lines, one was stimulated (NCI-H226), one was slightly inhibited (MGH-7) and three were not affected (NCI-H157, -H520 and -H1264). Both adenosquamous carcinoma (ADSQC) cell lines (MGH-30 and NCI-H125) were inhibited, but the effect on the two large-cell undifferentiated carcinoma (LCUC) cell lines (MGH-4, NCI-H66) was heterogeneous. As expected, the two cell lines that did not express HGFR/Met expression (NCI-H520 and -H661) also were not responsive to the mitogenic effect of HGF. The cell lines that were growth stimulated (MHG-13 and -4, NCI-H226) showed either low or intermediate Met/HGFR levels. In contrast, the majority of cell lines that were inhibited by HGF/SF showed high levels of Met/HGFR expression. Neither the *ras* genotype nor the presence of a putative HGF/SF autocrine loop appeared to influence the mitogenic responses of these cells to exogenous HGF/SF.

### Motogenic and invasion effect of HGF/SF

Cells were assessed for scatter activity in low-density cultures, both spontaneously in routine medium and after treatment with

**Figure 3** The mitogenic effect of HGF/SF on 15 NSCLC cell lines. The effect on proliferation is measured by the incorporation of [$^3$H]thymidine into the acid insoluble fraction of the cellular DNA after incubation in medium containing various concentrations of HGF/SF. The results are presented as the per cent incorporation compared with control untreated cells, the values being the means with standard error of means of three to six separate assays. Dashed lines indicate the 100% incorporation level of the control cells

**Figure 4** A representative microphotographs demonstrating the spontaneous and HGF/SF induced scatter activities in NSCLC cell lines. The NCI-H125 cells (**A**) normally demonstrated insignificant spontaneous scatter activity, whereas the A549 cells (**B**) showed a slight degree of spontaneous scatter activity. The treatment with 10 ng $ml^{-1}$ HGF/SF resulted in marked scatter activities in both cell lines (**C** H125; **D** A549). The microphotographs were taken on Giemsa-stained fixed cells, with the bar representing approximately 100 μm

**Table 2** The spontaneous and HGF/SF induced motogenic activity of NSCLC cell lines on a plastic surface and in collagen gels

| | | | Cell motility phenotypes[a] | | | |
|---|---|---|---|---|---|---|
| | | | Scattering (plastic surface) | | Invasion (collagen gel) | |
| Ras genotype | Histotype | Cell line | Control | HGF/SF | Control | HGF/SF |
| Mutant | ADC | MGH-8 | + | ↑ | + | ↑ |
| | | A549 | + | ↑ | + | ↑ |
| | | NCI-H358 | 0 | + | + | ↑ |
| | ADSQC | MGH-30 | 0 | NC | 0 | NC |
| | SQCC | NCI-H157 | + | NC | + | NC |
| | | NCI-H1264 | + | NC | + | NC |
| Wild type | ADC | MGH-13 | 0 | NC | 0 | + |
| | | MGH-24 | 0 | NC | 0 | NC |
| | | RVH-6849 | + | ↑ | 0 | + |
| | ADSQC | NCI-H125 | 0 | + | 0 | + |
| | SQCC | MGH-7 | 0 | NC | 0 | NC |
| | | NCI-H226 | 0 | + | + | ↑ |
| | | NCI-H520 | 0 | NC | + | NC |
| | LCUC | MGH-4[b] | 0 | NC | NA | NT |
| | | NCI-H661 | 0 | NC | 0 | NC |

[a]When tumour cell lines cultured at colony-forming densities or in collagen gels demonstrated individual cell dispersion, they were scored to show scattering or invasion phenotypes. When these phenomena were noted in the absence of HGF/SF, they were designated as spontaneous activity (+) under the control columns. After treatment with HGF/SF, these activities were either induced (+) or enhanced (↑). If the activities were similar before and after HGF/SF treatment, they were designated as NC (no change). [b]MGH-4 cell line did not form colonies in collagen gel, hence was not evaluated for its invasive property in collagen gel. NA, not assessed. The cell lines were segregated according to their ras genotypes and histogenesis. SQCC, squamous cell carcinoma; ADSQC, adenosquamous carcinoma; ADC, adenocarcinoma; LCUC, large cell undifferentiated carcinoma.

10 ng $ml^{-1}$ HGF/SF. Scatter activity was considered absent when tumour cells grew only as tight colonies (Figure 4A), but was considered present when some cells dispersed individually between colonies (Figure 4B–D). When compared with the untreated cells, the HGF/SF-induced scatter activity was scored as either induced, enhanced or unchanged (Table 2). Five cell lines showed varying degrees of spontaneous motility, and it was particularly marked in NCI-H157 and NCI-H1264. Four cell lines with spontaneous scatter activity had a mutation in their Ki-*ras* gene (Table 2). Only one of the nine cell lines with wild-type *ras* genes showed spontaneous scatter activity. The difference was statistically significant (Table 3), indicating a correlation between

 © Cancer Research Campaign 1998

**Table 3** The influence of *ras* genotype and autocrine HGF/SF loop in NSCLC cell lines on their spontaneous or HGF/SF induced/enhanced cell motility on a plastic surface

| | Spontaneous motility | | |
|---|---|---|---|
| | Absent | Present | *P*-value |
| *Ras* genotype | | | |
| Wild type | 8 | 1 | 0.011 |
| Mutant | 2 | 4 | |
| HGF/SF autocrine loop | | | |
| Absent | 4 | 3 | 0.608 |
| Present | 6 | 2 | |
| | **HGF/SF induced/enhanced motility** | | |
| | Absent | Present | *P*-value |
| *Ras* genotype | | | |
| Wild type | 6 | 3 | 0.622 |
| Mutant | 3 | 3 | |

*P*-values were evaluated using Fisher's exact test, two-tailed.

oncogenic *ras* activation and spontaneous motogenic activity in NSCLC cells. In contrast, there was a lack of correlation between the occurrence of spontaneous cellular motility and the presence of a putative HGF/SF autocrine loop (Table 3).

Treatment with 2 ng $ml^{-1}$ or higher concentrations of HGF/SF induced or enhanced cell scattering in six NSCLC cell lines (MGH-8, A549, RVH-6849, NCI-H125, -H226 and -H358). In two other lines (NCI-H157 and NCI-H1264) that demonstrated the greatest spontaneous motility, further stimulation of scatter activity by HGF/SF could not be evaluated. Both lines with no detectable HGFR/Met levels (NCI-H520 and -H661) also did not show HGF/SF-induced scattering. The ability of HGF/SF to induce or enhance cell motility in these NSCLC cells and the degree of such effects was not influenced by the *ras* genotype or the levels of Met/HGFR on the cells (Table 3).

The ability of these tumour cells to invade and migrate as single cells inside collagen gels was also evaluated (Figure 5 and Table 2). Four cell lines that demonstrated varying degrees of spontaneous scatter activity (MGH-8, A-549, NCI-H157 and -H1264) in monolayer culture, also showed varying degrees of spontaneous single cell invasion activity in collagen gels. Three other cell lines (NCI-H358, -H226 and -H520) additionally demonstrated a slight degree of such activity. HGF/SF induced or enhanced the collagen gel invasion activity in seven cell lines (MGH-8, A549, NCI-H358, MGH-13, NCI -H125, NCI-H226 and RVH-6849). There was an excellent correlation between the ability of HGF/SF to induce these cells to scatter on a plastic surface and in collagen gels.

### Morphogenic effect of HGF/SF

In collagen gels, all SQCC cell lines form irregular cell clusters, whereas most of ADC cell lines formed either round or rod-shaped cell aggregates (Figure 6A), which were composed of solid cords of cells without lumens formation (Figure 5A and C). After the addition of HGF/SF, none of the SQCC cell lines demonstrates morphogenic activity, whereas the cell aggregates of five ADC cell lines (A549, NCI-H358, MGH-30, MGH-13 and RVH-6849)

**Figure 5** The ability of NSCLC cells to demonstrate single-cell invasion or locomotion in collagen gels. Cells were seeded in 2.0 mg $l^{-1}$ collagen type I gels and allowed to form colonies spontaneously (**A** and **C**). After colonies have formed, duplicate wells were treated with 10 ng $l^{-1}$ HGF/SF (**B** and **D**). After a further 3–4 days' incubation, the gels were removed from the plates and fixed in 10% buffered formalin solution, embedded in paraffin, and sections were prepared and stained with haematoxylin and eosin. RVH-6849 cells formed tight colonies in control untreated gels (**A**) but demonstrated single-cell scattering and invasion after treatment with HGF/SF (**B**). In contrast, NCI-H358 cells demonstrated a slight degree of spontaneous single cells invasion (**C**), but this activity was markedly enhanced after treatment with HGF/SF (**D**). The bar lengths represent approximately 50 μm

© Cancer Research Campaign 1998

**Figure 6** The 'morphogenic activity' of NSCLC cells in collagen gels. MGH-30 cells formed round colonies (**A**) when grown in collagen gels but demonstrated irregular branchings of these cell aggregates after treatment with HGF/SF (**B**). The bar lengths represent approximately 50 μm showed varying degrees of and irregular branching extensions (Figure 6B). Histological sections, however, failed to show significant lumen formations in these branching cell cords. The ability to show 'branching extensions' was not influenced by the *ras* genotype of these cell lines.

### DISCUSSION

We have investigated the mitogenic, motogenic and morphogenic properties of fifteen NSCLC cell lines with known *ras* genotype and their responses to exogenous treatment with recombinant human HGF/SF. The results indicated that HGF/SF may have significant motogenic and morphogenic effects on these lung cancer cells, but a growth stimulatory effect occurred in only a minority of these cell lines.

Recombinant human HGF/SF exerted heterogeneous mitogenic effects on these cell lines, with six (40%) lines showing no response, and six others (40%) showing an inhibitory effect. Only three cell lines (20%) demonstrated significant growth stimulatory effect. Interestingly, the effect of HGF/SF on the proliferation of lung cancer cell lines has not been extensively reported, and ours represents the most comprehensive study on this subject. Among the four NSCLC cell lines that were studied and reported previously (Tajima et al, 1992; Yoshinaga et al, 1992; Singh-Kaw et al, 1995), HGF/SF stimulated growth in two lines (SQCC and LCUC respectively), and was ineffective in two others. None of these lines was derived from ADC. It is possible that the paucity of reports on the growth stimulatory effect of HGF/SF on lung cancer cells may actually reflect the infrequence of such findings, and this would be consistent with our results. The mitogenic effect of HGF/SF on other human tumour cell lines are very heterogeneous. HGF/SF inhibits the proliferation of all seven human hepatocellular carcinoma cell lines studied (Shiota et al, 1992), demonstrates no significant mitogenic effect on four out of five gastric carcinoma cell lines (Tanafel et al, 1994; Nagamine et al, 1996), and stimulates the growth of three of five malignant melanoma cell lines (Kan et al, 1991; Halaban et al, 1992). In contrast, HGF/SF consistently stimulates the proliferation of normal human epithelial cells in cultures. These include primary cultures of hepatocytes (Nakamura et al, 1987), mammary (Niranjan et al, 1995), biliary (Strain et al, 1995) and bronchial (Tsao et al, 1993) epithelia, keratinocytes (Matsumoto et al, 1991) and melanocytes (Halaban et al, 1992). It is worth noting that the activation of overexpressed tyrosine kinase receptors often leads to a suppression of cell proliferation, independent of the activation of the $p21^{ras}$ and MAP kinase (Osterop et al, 1994). This may explain the inability of constitutive *ras* activation to block the HGF/SF induced inhibition of proliferation in some of these NSCLC cell lines.

Our results have largely confirmed the prominent motogenic effect of HGF/SF on epithelial cells cultured on plastic surface and in collagen gels, including neoplastic cells. Among thirteen Met/HGFR expressing NSCLC cell lines that we studied, HGF/SF induced or enhanced scattering in six lines. Two other lines already demonstrated high levels of spontaneous cellular motility that precluded the evaluation of further enhancement. There is a very good correlation between the ability of these tumour cells to move on a plastic surface and in collagen gels, suggesting that the primary effect of HGF/SF is on cell movement, regardless of the environment of these cells. Recent reports suggested that the binding and activation of PI3-kinase, but not Grb-2/Sos-Ras pathways is important in HGF/SF-induced cell motility in MDCK cells (Fournier et al, 1996; Ponzetto et al, 1996). This would be consistent with our findings that the ability of HGF/SF to induce motogenic activity is not affected by the constitutive activation of the *ras* signal transduction pathway. Despite an absence of correlation between the presence of HGF/SF autocrine loop and spontaneous scatter activity, an autocrine motility function of HGF/SF in some of these NSCLC cells cannot be completely excluded. This requires further studies that use techniques to silence the HGF/SF expression and/or function. Recent reports indicated that a significant proportion of primary NSCLC overexpress HGF/SF and Met/HGFR (Harvey et al, 1996; Olivero et al, 1996; Takanami et al, 1996), and high levels of HGF/SF in these tissues may be correlated with a poor overall survival rate (Siegfried et al, 1997). The most important biological process that affects the survival or prognosis of lung cancer patients is metastasis, and cellular motility is one of the critical cellular functions during metastasis.

The observation that oncogenic *ras* activation is correlated with the spontaneous motogenic activity supports a hypothesis that the *ras* oncogene may promote early metastasis in NSCLC. Several studies have demonstrated that *ras* mutations in ADC is a poor prognostic factor, especially for early-stage disease (Rodenhuis and Slebos, 1992; Sugio et al, 1992). These patients usually die of distant metastatic recurrences.

Another important function of HGF/SF-Met/HGFR interaction is in morphogenesis, especially in the formation of three-dimensional tubular/glandular structures (Montesano et al, 1991). This effect has been demonstrated in vitro in normal epithelial cells, including human and mouse mammary epithelial cells (Niranjan et al, 1995),

 © Cancer Research Campaign 1998

epithelial cell lines derived from normal human prostate (Brinkmann et al, 1995) and mouse bile ductular and pancreatic epithelial cells (Johnson et al, 1993; Jeffers et al, 1996). In these normal cells, these branching cords of cells demonstrate lumen formations, thus consistent with glandular differentiation. We have also observed HGF/SF induced 'branching extensions' when lung ADC cell lines were cultured in collagen gels, but these structures fail to show gland lumen morphogenesis. In contrast, these branching cell cords showed irregular extensions that are more consistent with promotion of cell movement or dispersion. There is actually very scant published evidence to indicate that the glandular morphogenic activity of HGF/SF is functionally and commonly maintained in neoplastic cells. Brinkmann et al (1995) studied the morphogenic activity of HGF/SF in 64 human carcinoma cell lines of various organs and reported significant morphogenic activities in only three of these lines. Among the six lung carcinoma cell lines they studied, HGF/SF demonstrated induction of morphogenic activity in two lines, but only one (LX-1) line showed significant 'alveolar'-like lumen formation. In fact, only the LX-1 cell line that has been transfected by and is autocrinely expressing HGF/SF showed this effect, which is lost when a neutralizing antibody to HGF/SF was added. This indicates that the parent LX-1 cells do not spontaneously form alveolar structures. The discrepancy between normal and cancer cells in their morphogenic responses to HGF/SF may be caused by post-receptor modification of the Met/HGFR signal transduction pathways, or by alteration in the cytoskeletal filament organization in neoplastic cells.

### ACKNOWLEDGEMENT

This work is supported by grant 6191 from the Canadian Cancer Society and the National Cancer Institute of Canada.

### REFERENCES

Bottaro DP, Rubin JS, Faletto DL, Chan AM-L, Kmiecik TE, Vande Woude GF and Aaronson SA (1991) Identification of the hepatocyte growth factor receptor as the *c-met* prot-oncogene product. *Science* **251**: 802–804

Bussolino F, Di Renzo MF, Ziche M, Bocchietto E, Olivero M, Naldini L, Gaudino G, Tamagnone L, Coffer A and Comoglio PM (1992) Hepatocyte growth factor is a potent angiogenic factor which stimulates endothelial cell motility and growth. *J Cell Biol* **119**: 629–641

Brinkmann V, Foroutan H, Sachs M, Weidner KM and Birchmeier W (1995) Hepatocyte growth factor/scatter factor induces a variety of tissue-specific morphogenic programs in epithelial cells. *J Cell Biol* **131**: 1573–1586

Di Renzo MF, Narsimhan RP, Olivero M, Bretti S, Giordano S, Medico E, Gaglia P, Zara P and Comoglio PM (1991) Expression of Met/HGF receptor in normal and neoplastic human tissues. *Oncogene* **6**: 1997–2003

Fournier TM, Kamikura D, Teng K & Park M (1996) Branching tubulogenesis but not scatter of Mardin-Darby Canine Kidney cells requires a functional Grb2 binding site in the Met receptor tyrosine kinase. *J Biol Chem* **271**: 22211–22217

Gazdar AF and Oie HK (1986) Correspondence re: Martin Brower et al. Growth of cell lines and clinical specimens of human non-small cell lung cancer in a serum-free defined medium. *Cancer Res* **46**: 6011–6012

Grant DS, Kleinman HK, Goldberg ID, Bhargava MM, Nickoloff BJ, Kinsella JL, Polverini P & Rosen EM (1993) Scatter factor induces blood vessel formation in vivo. *Proc Natl Acad Sci USA* **90**: 1937–1941

Graziani A, Gramaglia D, Cantley LC & Comoglio PM (1991) The tyrosine-phosphorylated hepatocyte growth factor/scatter receptor associates with phosphatidylinositol 3-kinase. *J Biol Chem* **266**: 22087–22090

Graziani A, Gramaglia D, della Zonca P & Comoglio PM (1993) Hepatocyte growth factor/scatter factor stimulates the Ras-guanine nucleotide exchanger. *J Biol Chem* **268**: 9165–9168

Halaban R, Rubin JS, Funasaka Y, Cobb M, Boulton T, Faletto D, Rosen E, Chan A, Yoko K, White W, Cook C & Moellmann G (1992) Met and hepatocyte growth factor/scatter factor signal transduction in normal melanocytes and melanoma cells. *Oncogene* **7**: 2195–2206

Harvey P, Warn A, Newman P, Perry LJ, Ball RY & Warn RM (1996) Immunoreactivity for hepatocyte growth factor/scatter factor and its receptor, met, in human lung carcinomas and malignant mesothelioma. *J Pathol* **180**: 389–394

Jeffers M, Rao MS, Rulong S, Reddy JK, Subbarao V, Hudson E, Vande Woude GF & Resau JH (1996) Hepatocyte growth factor/scatter factor-Met signaling induces proliferation, migration, and morphogenesis of pancreatic oval cells. *Cell Growth Differ* **7**: 1805–1813

Johnson M, Koukoulis G, Matsumoto K, Nakamura T and Iyer A (1993) Hepatocyte growth factor induces proliferation and morphogenesis of nonparenchymal epithelial liver cells. *Hepatology* **17**: 1052–1061

Kan M, Zhang G, Zarnegar R, Michalopoulos G, Myoken Y, McKeehan WL and Stevens JI (1991) Hepatocyte growth factor/hepatopoietin A stimulates the growth of rat kidney proximal tubular epithelial cells (RPTE), rat nonparenchymal liver cells, human melanoma cells, mouse keratinocytes and stimulates anchorage-independent growth of SV-40 transformed RPTE. *Biochem Biophys Res Commun* **174**: 331–337

Liu C and Tsao M-S (1993*a*) *In vitro* and *in vivo* expressions of transforming growth factor-α and tyrosine kinase receptors in human non-small cell lung carcinomas. *Am J Pathol* **142**: 1155–1162

Liu C and Tsao M-S (1993*b*). Proto-oncogene and growth factor/receptor expression in the establishment of primary human non-small cell lung carcinoma cell lines. *Am J Pathol* **142**: 413–423

Longati P, Bardelli A, Ponzetto C, Naldini L and Comoglio PM (1994) Tyrosines$^{1234-1235}$ are critical for activation of the tyrosine kinase encoded by the *Met* proto-oncogene (HGF receptor). *Oncogene* **9**: 49–57

Matsumoto K and Nakamura T (1996) Emerging multipotent aspects of hepatocyte growth factor. *J Biochem* **119**: 591–600

Matsumoto K, Hashimoto K, Yoshikawa K and Nakamura T (1991) Marked stimulation of growth and motility of human keratinocytes by hepatocyte growth factor. *Exp Cell Res* **196**: 114–120

Matsumoto K, Matsumoto K, Nakamura T and Kramer RH (1994) Hepatocyte growth factor/scatter factor induces tyrosine phosphorylation of focal adhesion kinase ($p125^{FAK}$) and promotes migration and invasion by oral squamous cell carcinoma cells. *J Biol Chem* **269**: 31807–31813

Miyazawa K, Shimomura T, Kitamura A, Kondo J, Morimoto Y and Kitamura N (1993) Molecular cloning and sequence analysis of the cDNA for a human serine protease responsible for activation of hepatocyte growth factor. *J Biol Chem* **268**: 10024–10028

Montesano R, Schaller G and Orci L (1991) Induction of epithelial tubular morphogenesis in vitro by fibroblast-derived soluble factors. *Cell* **66**: 697–711

Nagamine K, Shibamoto S, Takeuchi K, Miyazawa K, Kitamura N, Chatani Y, Kohno M and Ito F (1996) Dissociation of c-fos induction and mitogen-activated-protein kinase activation from the hepatocyte-growth-factor-induced motility response in human gastric carcinoma cells. *J Biochem* **236**: 476–481

Nakamura T, Nawa K, Ichihara A, Kaise N and Nishino T (1987) Purification and subunit structure of hepatocyte growth factor from rat platelets. *FEBS Lett* **224**: 311–316

Naldini L, Tamagnone L, Vigna E, Sachs M, Hartmann G, Birchmeir W, Daikuhura Y, Tsubouchi H, Blasi F and Comoglio PM (1992) Extracellular proteolytic cleavage by urokinase is required for activation of hepatocyte growth factor. *EMBO J* **11**: 4825–4833

Niranjan B, Buluwela L, Yant J, Perushinghe N, Atherton A, Phippard D, Dale T, Gusterson B and Kamalati T (1995) HGF/SF: a potent cytokine for mammary growth, morphogenesis and development. *Development* **121**: 2897–2908

Okano Y, Mizuno K, Osada S, Nakamura T and Nozawa Y (1993) Tyrosine phosphorylation of phospholipase Cγ in *c-met*/HGF receptor-stimulated hepatocytes: comparison with HepG2 hepatocarcinoma cells. *Biochem Biophys Res Commun* **190**: 842–848

Olivero M, Rizzo M, Madeddu R, Casadio C, Pannacchietti S, Nicotra MR, Prat M, Maggi G, Arena N, Natali PG, Comoglio PM and Di Renzo MF (1996) Overexpression and activation of hepatocyte growth factor/scatter factor in human non-small-cell lung carcinomas. *Br J Cancer* **74**: 1862–1868

Osterop APRM, Medema RH, Ouwens DM, Van der Zon GCM, Moller W and Maasen JA (1994) Activation of overexpressed receptors for insulin and epidermal growth factor interferes in mitogenic signaling without affecting the activation of p21ras. *Biochemistry* **33**: 7453–7459

Park M, Dean M, Cooper CS, Schmidt M, O'Brien SJ, Blair DG and Vande Woude GF (1986) Mechanism of *met* oncogene activation. *Cell* **45**: 895–904

Pelicci G, Giordano S, Zhen Z, Salcini AE, Lanfrancone L, Bardelli A, Panayotou G, Waterfield MD, Ponzetto C, Pelicci PG and Comoglio PM (1995) The motogenic and mitogenic responses to HGF are amplified by the Shc adaptor protein. *Oncogene* **10**: 1631–1638

© Cancer Research Campaign 1998 

Phelps RM, Johnson BE, Ihde DC, Gazdar AF, Carbone DP, McClintock PR, Linnoila RI, Matthews MJ, Bunn PA Jr, Carney D, Minna JD and Mulshine JL (1996) NCI-Navy medical oncology branch cell line data base. *J Cell Biochem* **24** (suppl.): 32–91

Ponzetto C, Bardelli A, Zhen Z, Maina F, della Zonca P, Giordano S, Grazini A, Panayotou G and Comoglio PM (1994) A multifunctional docking site mediates signaling and transformation by the hepatocyte growth factor/scatter factor receptor family. *Cell* **77**: 261–271

Ponzetto C, Zhen Z, Audero E, Maina F, Bardelli A, Basile ML, Giordano S, Narshimhan R and Comoglio P (1996) Specific uncoupling of GRB2 from the Met receptor. *J Biol Chem* **271**: 14119–14123

Rodenhuis S and Slebos RJC (1992) Clinical significance of ras oncogene activation in human lung cancer. *Cancer Res* **52**: 2665s–2669s

Rosen EM, Nigam SK and Goldberg ID (1994) Scatter factor and the c-met receptor: a paradigm for mesenchymal/epithelial interaction. *J Cell Biol* **127**: 1783–1787

Royal I and Park M (1995) Hepatocyte growth factor-induced of Madin-Darby Canine Kidney cells requires phosphatidylinositol 3-kinase. *J Biol Chem* **270**: 27780–27787

Rygaard K, Nakamura T and Spang-Thomsen M (1993) Expression of the proto-oncogenes c-*met* and c-*kit* and their ligands, hepatocyte growth factor/scatter factor and stem cell factor, in SCLC cell lines and xenografts. *Br J Cancer* **67**: 37–46

Shiota G, Rhoads DB, Wang TC, Nakamura T and Schmidt EV (1992) Hepatocyte growth factor inhibits growth of hepatocellular carcinoma cells. *Proc Natl Acad Sci USA* **89**: 373–377

Siegfried JM, Weissfeld LA, Singh-Kaw P, Weyant RJ, Testa JR and Landreneau RJ (1997) Association of immunoreactive hepatocyte growth factor with poor survival in resectable non-small cell lung cancer. *Cancer Res* **57**: 433–439

Singh-Kaw P, Zarnegar R and Siegfried JM (1995) Stimulatory effects of hepatocyte growth factor on normal and neoplastic human bronchial epithelial cells. *Am J Physiol* **268**: L1012–1020

Stoker M, Gheraldi E, Perryman M and Gray J (1987) Scatter factor is a fibroblast-derived modulator of epithelial cell mobility. *Nature* **327**: 239–242

Strain AJ, Wallace L, Joplin R, Daikuhara Y, Ishii T, Kelly DA and Neuberger JM (1995) Characterization of biliary epithelial cells isolated from needle biopsies of human liver in the presence of hepatocyte growth factor. *Am J Pathol* **146**: 537–545

Sugio K, Ishida T, Yokoyama H, Inoue T, Sugimachi K and Sazuki T (1992) Ras gene mutation as a prognostic marker in adenocarcinoma of human lung without lymph node metastasis. *Cancer Res* **52**: 2903–2906

Tajima H, Matsumoto K and Nakamura T (1992) Regulation of cell growth and motility by hepatocyte growth factor and receptor expression in various cell species. *Exp Cell Res* **202**: 423–431

Takanami I, Tanana F, Hashizume T, Kikuchi K, Yamamoto Y, Yamamoto T and Kodaira S (1996) Hepatocyte growth factor and c-met/hepatocyte growth factor receptor in pulmonary adenocarcinomas: an evaluation of their expression as prognostic markers. *Oncology* **53**: 392–397

Tanapfel A, Yasui W, Yokozaki H, Wittekind C and Tahara E (1994) Effect of hepatocyte growth factor on the expression of E- and P-cadherins in gastric carcinoma cell lines. *Virchows Arch* **425**: 139–144

Tsao M-S, Zhu H, Giaid A, Viallet J, Nakamura T and Park M (1993) Hepatocyte growth factor/scatter factor is an autocrine factor for human normal bronchial epithelial and lung carcinoma cells. *Cell Growth Different* **4**: 571–579

Tsao M-S, Liu N, Nicklee T, Shepherd F and Viallet J (1997) Angiogenesis correlates with vascular endothelial growth factor expression but not with Ki-ras oncogene activation in non-small cell lung carcinoma. *Clin Cancer Res* (in press)

Weidner KM, Behrens J, Vandekerchhove J and Birchmeier W (1990) Scatter factor: molecular characteristics and effect on the invasiveness of epithelial cells. *J Cell Biol* **111**: 2097–2108

Weidner KM, Arakaki N, Hartmann G, Vandekerckhove J, Weingart S, Rieder H, Fontatsch C, Tsubouchi H, Hishida T, Daikuhara Y and Birchmeir W (1991) Evidence for the identity of human scatter factor and human hepatocyte growth factor. *Proc Natl Acad Sci USA* **88**: 7001–7005

Yoshinaga Y, Fujita S, Gotoh M, Nakamura T, Kikuchi M and Hirohashi S (1992) Human lung cancer cell line producing hepatocyte growth factor/scatter factor. *Jpn J Cancer Res* **83**: 1254–1261

Zhu H, Naujokas MA and Park M (1994*a*) Receptor chimeras indicate that the Met tyrosine kinase mediates the motility and morphogenic responses of hepatocyte growth factor/scatter factor. *Cell Growth Different* **5**: 359–366

Zhu H, Naujokas MA, Fixman ED, Torossian K and Park M (1994*b*) Tyrosine 1356 in the carboxyl-terminal tail of the HGF/SF receptor is essential for the transduction of signals for cell motility and morphogenesis. *J Biol Chem* **269**: 29943–29948

Zioncheck TF, Richardson L, Liu J, Chang L, King KL, Bennett GL, Fugedi P, Chamow SM, Schwall R and Stack RJ (1995) Sulfated oligosaccharides promote hepatocyte growth factor association and govern its motogenic activity. *J Biol Chem* **270**: 16871–16878

 © Cancer Research Campaign 1998